# The Interaction of Single Nucleotide Polymorphisms on Fibroblast Growth Factor 19 Superfamily Genes Is Associated With Alcohol Dependence-Related Aggression

**DOI:** 10.3389/fgene.2021.695835

**Published:** 2021-08-18

**Authors:** Jinzhong Xu, Fenzan Wu, Fan Wang, Fan Yang, Meng Liu, Mengbei Lou, Linman Wu, Hui Li, Wenhui Lin, Yunchao Fan, Li Chen, Yanlong Liu, Haiyun Xu, Jue He

**Affiliations:** ^1^Department of Clinical Pharmacy, Affiliated Wenling Hospital, Wenzhou Medical University, Wenling, China; ^2^Laboratory of Translational Medicine, Affiliated Cixi Hospital, Wenzhou Medical University, Ningbo, China; ^3^Psychiatry Research Center, Beijing Hui-Long-Guan Hospital, Peking University, Beijing, China; ^4^Xinjiang Key Laboratory of Neurological Disorder Research, The Second Affiliated Hospital of Xinjiang Medical University, Ürümqi, China; ^5^School of Mental Health, Wenzhou Medical University, Wenzhou, China; ^6^Department of Cardiovascular Medicine, Affiliated Wenling Hospital, Wenzhou Medical University, Wenling, China; ^7^The Affiliated Kangning Hospital, Wenzhou Medical University, Wenzhou, China; ^8^Xiamen Xian Yue Hospital, Xiamen, China; ^9^First Affiliated Hospital, Institute of Neurological Disease, Henan University, Kaifeng, China; ^10^Institute of Aging, Key Laboratory of Alzheimer’s Disease of Zhejiang Province, Wenzhou Medical University, Wenzhou, China

**Keywords:** FGF21, FGF19, FGF23, alcohol dependence, aggression, single nucleotide polymorphism

## Abstract

Alcohol dependence (AD) is characterized by compulsive alcohol consumption, which involves behavioral impairments such as aggression. Members of fibroblast growth factor (FGF) 19 superfamily, including FGF19, FGF21, and FGF23, are major endocrine mediators that play an important role in alcohol metabolism and alcohol related disorders. The objective of the present study is to explore the possible associations among the interaction of single nucleotide polymorphisms (SNPs) of the FGF 19 superfamily, AD occurrence, and aggression in patients with AD. A total of 956 subjects were enrolled in this study, including 482 AD patients and 474 healthy controls (HCs). Michigan alcoholism screening test (MAST) was used to measure the level of AD, a Chinese version of the Buss–Perry Aggression Questionnaire was used to evaluate the aggressive behavior of subjects, and MassARRAY^@^ system was used to genotype rs948992 of FGF19, rs11665841 and rs11665896 of FGF21, rs7955866 and rs11063118 of FGF23. The results showed that AD patients presented a significantly higher level of aggression compared to HCs, and MAST scores were significantly positively associated Buss–Perry aggression scores (*r* = 0.402, *p* < 0.001) in AD patients. The interaction of FGF19 rs948992 TC × FGF21 rs11665896 GG presented the high-risk genotype combination predicting the high level of AD. In addition, the interaction of FGF19 rs948992 TC × FGF21 rs11665896 TG × FGF23 rs11063118 TT presented the high-risk genotype combination predicting the high level of aggression in AD patients. Our results added evidence linking the combination of rs948992 TC × rs11665896 TG × rs11063118 TT to aggressive behavior in AD patients and pointed out the potential usefulness of the SNPs of FGF19 superfamily as a predictor for the aggression in AD patients.

## Introduction

Alcohol dependence (AD) is a common psychiatric disorder and is characterized by loss of control over alcohol drinking, which is associated with impaired decision-making, seeking alcohol regardless of health status and behavioral impairments such as aggression and impulsivity, even suicide attempts (Tobore, 2019; Witkiewitz et al., 2019). Pieces of evidence suggested that AD was a multifactorial and genetic disease, and the pathogenesis of AD included neurobiological, genetic and epigenetic, psychological, social, and environmental factors (Kiive et al., 2017; Newman et al., 2018; Malamut et al., 2021). Some patients with AD recover after lifestyle modifications in the absence of medical treatment, while many of them relapse and display damaged brain structure and function (Grant et al., 2015; Abrahao et al., 2017; Erickson et al., 2019). AD is usually reported to combine with behavioral impairments or mental disorders such as aggression, suicide, major depression, anxiety, insomnia, and drug addiction (Dick and Agrawal, 2008; Schuckit, 2009).

As a harmful behavior induced by AD, aggression is an important alcohol-related phenotype and could endanger other individuals and society in general through violence and even crime (Heinz et al., 2011; Chester et al., 2020). Increasing evidence suggested that neurobiological, genetic and epigenetic mechanisms such as the involvement of dopamine, serotonin, gamma-aminobutyric acid (GABA), and neuroendocrine systems may be related to AD and AD-related aggression (Heinz et al., 2011; Nedic Erjavec et al., 2014; Gan et al., 2015; Plemenitas et al., 2015; Waltes et al., 2016). The environmental factors and specific genes may be the important causes for AD and alcohol-use disorders including aggression (Schuckit, 2009; Plemenitas et al., 2015). The interaction among the single nucleotide polymorphisms (SNPs) of genes of oxytocin and its receptor was reported to be related to alcohol disorder and aggressive behavior (Yang et al., 2017). The GABA type A receptor subunit alpha (GABRA) 2 rs279826/rs279858 A-allele interacts with stress, contributing to the development of alcohol use and aggressive behavior (Kiive et al., 2017).

Fibroblast growth factor (FGF) 19, FGF21, and FGF23 belong to FGF19 superfamily. The members of this subfamily are characterized by their reduced binding affinity for heparin that enables them to be transported in the circulation and function in an endocrine manner (Kharitonenkov and DiMarchi, 2017). The proteins of FGF19 subfamily influence the enterohepatic circulation of bile, participate in glucose and lipid metabolism regulation, and maintenance of phosphorus and vitamin D3 homeostasis (Dolegowska et al., 2019). Recent studies suggest that FGF19, FGF21, and FGF23 are associated with alcohol and alcohol-related disorders (Schumann et al., 2016; Quintero-Platt et al., 2017; Brandl et al., 2018; Gonzalez-Reimers et al., 2018; Epperlein et al., 2021). Serum FGF19 level and FGF19 mRNA expression both increase strongly in patients with alcohol use disorder (Brandl et al., 2018). The circulating FGF 21 level also increased significantly after binge alcohol consumption (Desai et al., 2017). Studies suggested heavy alcohol intake could induce FGF 21 high expression, however, overexpressing FGF 21 may decrease alcohol preference by regulating the drinking behavior in part through SIM1-positive neurons of the hypothalamus (Desai et al., 2017; Soberg et al., 2018; Song et al., 2018). In alcoholic patients, the levels of FGF 23 also increased significantly (Quintero-Platt et al., 2017). The evidence about FGF19 superfamily members associated with alcohol consumption is relatively abundant, but the reports about FGF19 superfamily members’ polymorphisms with AD are still rare. A study found that the A allele of rs838133 on the FGF21 gene is significantly associated with increased alcohol intake (Soberg et al., 2017). Another study demonstrated that the FGF19/21-β-Klotho signaling pathway is associated with alcohol consumption and the substream essential receptor protein β-Klotho gene SNP rs11940694 is significantly related to alcohol intake (Schumann et al., 2016).

As an important member of the FGF19 superfamily, FGF21 has an extensive biologic effect on mediating the central nervous system. FGF21 might be involved in mood disorders through stimulating dopamine signaling in the prefrontal cortex (Chiavaroli et al., 2017). A recent study has indicated that FGF21 was significantly associated with alcohol craving (Epperlein et al., 2021), which is associated with aggression in patients with alcohol use disorder (Roozen et al., 2013; Park et al., 2019). FGF21 genetic or near the FGF21 locus variants are associated with carbohydrate preference and consumption of addictive substances (Heianza et al., 2016; Frayling et al., 2018). Furthermore, physiological effects of FGF21 were tightly correlated with the dopamine and serotonin system (Han et al., 2016; Recinella et al., 2017; Gibson, 2018), which both play an important role in mediating aggressive behavior (Brodie et al., 2016). All the above studies indicate a potential association between FGF19 superfamily and aggressive behavior.

The rs948992 of FGF19 SNP and rs11665841 and rs11665896 of FGF21 SNPs are located on 3′ untranslated region (3′UTR; Zhang et al., 2012),^1^ where miRNAs could bind and further impact target gene protein translation (Chen and Rajewsky, 2006; Landi et al., 2008). The genetic variants of miRNA target sites may induce phenotypic changes and increase the risk of developing diseases (Sethupathy and Collins, 2008; Zhang et al., 2012). The sequence variation C716T (rs7955866) is located in exon 3 of FGF23 gene, which could affect the FGF23 activity by the missense variation designated T239M and the exchange of T239M (Nguyen et al., 2010). In addition, rs11063118 of FGF23 was selected based on the tagging SNP approach, optimal sets of SNPs were derived from genetic databases in order to tag haplotypes across genes (Rothe et al., 2017). Thus, in order to explore the possible associations of FGFs 19, 21, and 23 polymorphisms with AD and AD-related aggression, rs948992 of FGF19, rs11665841, and rs11665896 of FGF21, and rs7955866 and rs11063118 of FGF23 were chosen to evaluate genotypes by using MassARRAY^@^ system, Michigan alcoholism screening test (MAST) was used to measure the level of AD, and a Chinese version of the Buss–Perry Aggression Questionnaire was used to evaluate the aggressive behavior of subjects in the present study.

## Materials and Methods

### Subjects

A total of 956 unrelated Han population males aged above 18 years in northern China were recruited in this cross-section study, including 482 patients in AD-group and 474 healthy control subjects in HC-group. Because there were too few female patients with AD in this study, they were excluded. All alcoholism patients were diagnosed according to the Diagnostic and Statistical Manual of the American Psychiatric Association, fourth edition (DSM-IV). Participants with the following conditions were excluded: had a family history of psychosis and neurological diseases, severe systemic diseases, central nervous system diseases, cancer, and cognitive impairment. According to self-reporting and confirmation by the next of kin and family members, subjects with a history of drug abuse or dependence were also excluded, except for alcohol and nicotine abuse. The clinical characteristics data were collected after enrollment including age, years of education, marital status, and living conditions.

### The Assessment of Aggression Associated With Alcohol Dependence

The Chinese version (Yang et al., 2017) revised from Buss–Perry Aggression Questionnaire (Buss and Perry, 1992) was used to measure five aspects of human aggression. The original 29-item scale with 4 subscales became a 30-item scale with 5 subscales: physical aggression (7 items), verbal aggression (5 items), anger (6 items), hostility (7 items), and aggression toward self (5 items). All items are 5-point Likert scales (none, seldom, sometimes, often, and always) scored 0–4, so the total score ranges from 0 to 120 with higher scores representing greater aggression. In addition, the MAST was performed to evaluate the influence of alcohol on individuals with AD (Selzer, 1971). The MAST is a 25-item, self-report questionnaire on which respondents rate the severity of a range of AD using a 4-point scale ranging from 1 (not at all) to 4 (extremely). The scale has high internal-consistency reliability, with alpha values of 0.90 (Skinner, 1982). Higher scores indicate greater AD. This study was approved by the Peking University Institutional Review Board. For each participant, written informed consent was obtained directly from the subjects or their responsible guardians. All procedures performed in this study involving human participants were following the 1964 Helsinki declaration and its later amendments or comparable ethical standards.

### Selection and Genotyping of Single Nucleotide Polymorphism

Genomic DNA was extracted from 5 ml peripheral blood using the salting-out method from all the subjects (Tian et al., 2008). Buffy coats of nucleated cells obtained from anticoagulated blood (ACD or EDTA) were resuspended in 15 ml polypropylene centrifugation tubes with 3 ml of nuclei lysis buffer (10 mM Tris–HCl, 400 mM NaCl, and 2 mM Na_2_EDTA, pH 8.2). The cell lysates were digested overnight at 37°C with 0.2 ml of 10% SDS and 0.5 ml of a protease K solution (1 mg protease K in 1% SDS and 2 mM Na_2_EDTA). After digestion was complete, 1 ml of saturated NaCl (approximately 6M) was added to each tube and shaken vigorously for 15 s, followed by centrifugation at 2,500 rpm for 15 min. The precipitated protein pellet was left at the bottom of the tube and the supernatant containing the DNA was transferred to another 15 ml polypropylene tube. Exactly 2 volumes of room temperature absolute ethanol was added and the tubes inverted several times until the DNA precipitated. The precipitated DNA strands were removed with a plastic spatula or pipette and transferred to a 1.5 ml microcentrifuge tube containing 100–200 μl TE buffer (10 mM Tris–HCl, 0.2 mM Na_2_EDTA, pH 7.5). The DNA was allowed to dissolve 2 h at 37°C before quantitating (Miller et al., 1988). Three SNPs of the gene including rs948992, rs11665841, rs11665896, rs7955866, and rs11063118 were genotyped using MALDI-TOF based scalable MassARRAY^@^ System (Agena Bioscience, Inc., San Diego, CA, United States). The protocol was performed according to the manufacturer’s instructions. The primers are shown in Table 1. All the laboratory procedures were carried out in a blind manner to case/control status. The conditions of PCR were as follows: 94°C for 30 s [40 cycles × (94°C for 5 s), 5 cycles × (52°C for 5 s 80°C for 5 s, and 72°C for 3 min)]. Ten percent of the DNA samples were duplicated randomly and tested, and no-fault genotyping was found.

**TABLE 1:** Polymerase chain reaction (PCR) primers used for single nucleotide polymorphism (SNP) analysis of FGF19, FGF21, and FGF23 genes.

**Polymorphism**	**Primer**	**Sequence (5′–3′)**
rs948992	R-primer	ACGTTGGATGTAACTTGCTGTCCCGGTGTC
	F-primer	ACGTTGGATGAATCCATGGGGAGGCATGTG
	Single base extension	CAGAGGGCTGGTGGGCTGGG
rs11665841	R-primer	ACGTTGGATGAGAGCGAGACTCCGTCTCAA
	F-primer	ACGTTGGATGTACACCTCCCCTCACGTGG
	Single base extension	AAAAAGTGAGGCCCA
rs11665896	R-primer	ACGTTGGATGCAGCTGTTTTGTCTCCCTTG
	F-primer	ACGTTGGATGGAAAAAAGTGAGGCCCAGTG
	Single base extension	CCCATCCCCTCACGTGGTCC
rs11063118	R-primer	ACGTTGGATGGCTCACGTTTAATAGCTGGG
	F-primer	ACGTTGGATGAATGGGCAGTGCAGACTAGG
	Single base extension	TGGGGTTTGAACTCAGGCA
rs7955866	R-primer	ACGTTGGATGTCGAGTGAACACGCACGCTG
	F-primer	ACGTTGGATGAGCGACCCTAGATGAACTTG
	Single base extension	GGGCACACGCACGCTGGGGGAA

### Statistical Analysis

Continuous variables were expressed as the mean ± standard and categorical variables were expressed as the absolute value. χ^2^ test was performed to evaluate the difference of the categorical variables between the groups. Analysis of variance (ANOVA) for age and education years was performed to assess the differences of groups. Analysis of covariance (ANCOVA) for other continuous variables was performed with age and years of education as covariates using SPSS 20.0 (Statistical Package for Social Studies, Version 20.0, SPSS Inc., Chicago, IL, United States). Hardy--Weinberg equilibrium (HWE) test, linkage disequilibrium (LD) and the frequencies of the distribution of genotypes and alleles in both AD-group and HC-group were analyzed using SHEsis and SHEsis-Plus platform^2^ (Shi and He, 2005). Generalized multifactor dimensionality reduction (GMDR), a genetic model-free alternative to logistic regression, is used to detect the interaction of gene and environment, which was performed to calculate the interaction of five loci (Lou et al., 2007). All tests were two-tailed, the *p*-value less than 0.05 was considered statistically significant.

## Results

### The General Characteristics of the Study Population

In the present study as shown in Table 2, all the participants ranged from 20 to 67 years old (40.63 ± 11.68 years) with the education years from 5 to 18 years (10.78 ± 3.48 years). The age of subjects in the AD-group was 44.08 ± 11.96 years and significantly higher than that of the HC-group (*p* < 0.001), but the education years of subjects of the former were 9.88 ± 3.68 years and significantly lower than that of the latter (*p* < 0.001). In the AD-group, the proportion of patients with never married was 9.3% and was significantly lower compared with the HC-group (*p* < 0.001). The rate of patients with divorce/widowed history was significantly higher than that of the HC-group after multiple comparisons (*p* < 0.001). The MAST (Chinese version) 19 was performed to evaluate the severity of AD and it is a 22-item self-scoring test.

**TABLE 2:** Clinical characteristics of all participants.

**Characteristics**	**AD (*n* = 482)**	**HC (*n* = 474)**	***p***
Age	44.08 ± 11.96	37.07 ± 10.24	<0.001
Years of education	9.88 ± 3.68	11.70 ± 2.99	<0.001
**Marital status *n*, (%)**			
Never married	45 (9.3%)	105 (22.2%)	<0.001
Married	362 (75.1%)	352 (74.3%)	0.765
Divorced/widowed	75 (15.6%)	17 (3.6%)	<0.001
**Living conditions**			
Live alone	65 (13.5%)	87 (18.4%)	0.040
Living ingroup quarters	55 (11.4%)	58 (12.2%)	0.693
Livingwith family	362 (75.1%)	329 (69.4%)	0.049

*AD, alcohol dependences; HC, healthy controls.*

*Analysis of variance was performed to analyze the difference of age and years of education between the groups. χ^2^ test was performed to evaluate the difference of the categorical variables between the groups.*

### The Difference in Aggression Between the Two Groups

Because the age and years of education were different between the two groups, ANCOVA was used to calculate the difference of aggression with the year of education as covariables, respectively. The Aggression Scale total score was 34.83 ± 17.63 in the AD-group and higher than that of the HC-group (21.71 ± 15.04; *p* < 0.001). Physical aggression of the AD-group was higher compared with the HC-group (37.13 ± 21.01 vs. 26.15 ± 18.81; *p* < 0.001). Verbal aggression also significantly increased in the AD-group (41.41 ± 23.40 vs. 22.68 ± 18.94; *p* < 0.001). The level of anger in the AD-group was 41.41 ± 23.40 and significantly higher than that of the HC-group (*p* < 0.001). The subjects in the AD-group have stronger hostility than the HC-group (30.579 ± 19.18 vs. 18.12 ± 15.90; *p* < 0.001). The levels of self-aggression also elevated in subjects with AD than that of the HC-group (33.10 ± 20.05 vs. 15.01 ± 16.72; *p* < 0.001; Table 3).

**TABLE 3:** The difference in aggression related to AD compared with the HC-group.

**Aggression and MAST scores**	**AD-group (*n* = 482)**	**HC-group (*n* = 474)**	***p***
Aggression Scale total score	34.83 ± 17.63	21.71 ± 15.04	<0.001
Physical aggression	37.13 ± 21.01	26.15 ± 18.81	<0.001
Verbal aggression	37.72 ± 19.87	26.29 ± 18.15	<0.001
Anger	41.41 ± 23.40	22.68 ± 18.94	<0.001
Hostility	30.579 ± 19.18	18.12 ± 15.90	<0.001
Self-aggression	33.10 ± 20.05	15.01 ± 16.72	<0.001
MAST scores	10.09 ± 5.07	/	

*AD, alcohol dependences; HC, healthy controls.*

*Analysis of covariance was performed to analyze the difference between the two groups with years of education as covariates.*

### The Association Between Aggression and MAST Scores

**FIGURE 1:**
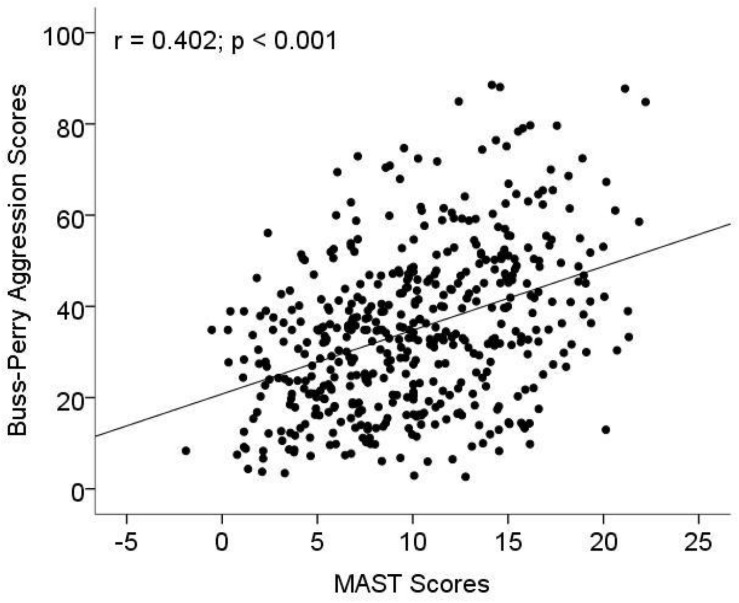
The association between aggression and Michigan alcoholism screening test (MAST) scores. MAST was used to measure the level of alcohol dependence (AD), and a Chinese version of the Buss–Perry Aggression Questionnaire was used to evaluate the aggressive behavior of subjects.

The association between MAST scores and aggression related to AD were investigated with age and years of education as covariables. The result showed that MAST scores were significantly positively associated Buss–Perry aggression scores (*r* = 0.402, *p* < 0.001; Figure 1). In multiple linear regression model, increased MAST scores contributed to Buss–Perry aggression scores (OR: 1.44, 95%CI: 1.1–1.75) after being adjusted for age, education years and marital status (Table 4).

**TABLE 4:** The multiple linear regression analysis of the association between MAST scores and Buss–Perry aggression scores.

**Buss–Perry aggression scores**	**Model 1**		**Model 2**	
	**β, 95% CI**	***p***	**β, 95% CI**	***p***
MAST scores	1.41 (1.11–1.70)	<0.001	1.44 (1.14–1.75)	<0.001

*Model 1: unadjusted.*

*Model 2: adjusted for age, education years and marital status.*

### Genetic Polymorphisms Analysis

Genotype distributions of rs948992, rs11665841, rs11665896, rs7955866, and rs11063118 loci were consistent with Hardy–Weinberg proportions (AUD: χ^2^ = 0.043, *p* = 0.98; χ^2^ = 1.81, *p* = 0.40; χ^2^ = 1.95, *p* = 0.38; χ^2^ = 1.47, *p* = 0.48; χ^2^ = 7.40, *p* = 0.024; HC: χ^2^ = 0.13, *p* = 0.94; χ^2^ = 3.12, *p* = 0.21; χ^2^ = 2.33, *p* = 0.31; χ^2^ = 0.46, *p* = 0.79; and χ^2^ = 2.17, *p* = 0.34, respectively). As shown in Table 5, the distributions of genotypes and alleles of the five loci were not significantly different between the two groups, respectively (all *p* > 0.05).

**TABLE 5:** The frequencies of genotypic and allelic distributions of five loci.

**SNP**		**Major/minor allele**	**Major allele frequency**		***p***	**Major allele frequency**		***p***
			**HC**	**AD**		**HC (AA/Aa/aa)**	**AD (AA/Aa/aa)**	
FGF19	rs948992	C/T	0.51	0.54	0.27	121/225/112	121/213/90	0.49
FGF21	rs11665841	C/T	0.68	0.69	0.76	230/187/55	219/177/48	0.93
	rs11665896	G/T	0.68	0.69	0.84	230/190/54	219/178/49	0.92
FGF23	rs7955866	G/A	0.84	0.85	0.52	337/123/14	327/106/13	0.76
	rs11063118	T/C	0.80	0.81	0.68	308/141/24	298/119/25	0.61

*AA = major allele homozygous; Aa = heterozygous; aa = minor allele.*

### Generalized Multifactor Dimensionality Reduction Analysis of Gene–Gene of FGF 19 and 21 Interaction for AD

**FIGURE 2:**
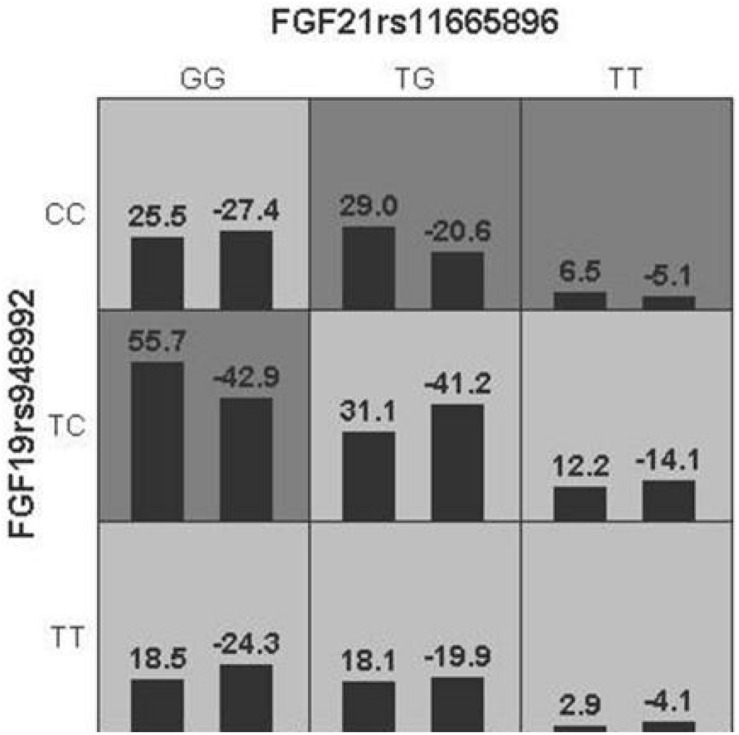
The best model based on generalized multifactor dimensionality reduction (GMDR) analyses showed the interaction effect of FGF19 rs948992 TC × FGF21 rs11665896 GG on AD. The shaded dark gray cell presented a high-risk combination and shaded light gray presented a low-risk combination.

Generalized multifactor dimensionality reduction analysis was performed to evaluate the cross-validation consistency and the prediction error for each number of loci. The model of FGF21 rs11665896 – FGF19 rs948992 combination was significant (*p* = 0.010) with a training accuracy of 55.68% and testing accuracy of 53.97%, and with a maximum cross-validation consistency (10/10) after permutation testing (Table 6). The two-locus genotype combinations related to different risk for each multilocus-genotype combination was analyzed and showed in Figure 2. The model of FGF21 rs11665896 (GG) – FGF19 rs948992 (TC) presented the high-risk genotype combination, and FGF21 rs11665896 (TT) – FGF19 rs948992 (TT) presented the low-risk genotype combinations (Figure 2).

**TABLE 6:** The best model for predicting the occurrence of AD.

**Best model^*a*^**	**Training accuracy (%)**	**Testing accuracy (%)**	**CVC**	**χ^2^**	***p***	**95% CI**
1	52.71	37.80	6/10	1.07	0.302	1.24 (0.82, 1.88)
1, 2	55.68	53.97	10/10	6.51	0.010	1.74 (1.13, 2.66)
1, 2, 3	57.26	51.21	5/10	0.0064	0.936	1.05 (0.29, 3.71)
1, 2, 3, 4	58.66	51.82	8/10	0.715	0.398	1.72 (0.48, 6.08)
1, 2, 3, 4, 5	59.37	50.71	10/10	1.004	0.316	1.97 (0.52, 7.50)

*^*a*^Number 1–5 represented FGF19 rs948992, FGF21 rs11665896, FGF23 rs11063118, FGF23 rs7955866, and FGF21 rs11665841, respectively.*

### Generalized Multifactor Dimensionality Reduction Analysis of Gene–Gene Interaction for Aggression Associated With AD

**FIGURE 3:**
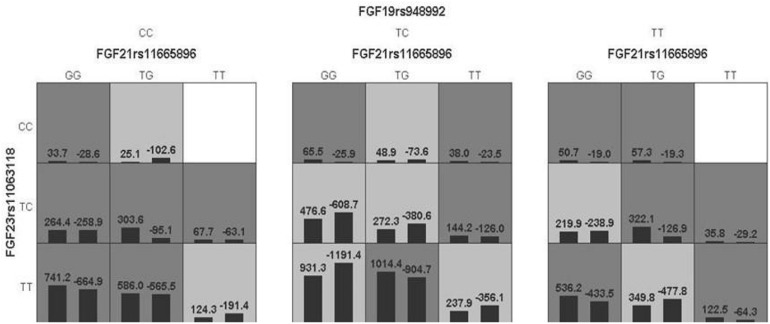
The best model based on GMDR analyses showed the interaction effect of FGF19 rs948992 TC × FGF21 rs11665896 TG × FGF23 rs11063118 TT on AD related aggression. The shaded dark gray cell presented a high-risk combination and shaded light gray presented a low-risk combination.

The model of FGF 19rs948992 – FGF21 rs11665896 – FGF23 rs11063118 combination was significant (*p* = 0.0017) with a training accuracy of 56.85% and testing accuracy of 50.32%, and with a close to maximum cross-validation consistency (9/10) after permutation testing (Table 7). The two-locus genotype combinations related to a different risk for each multilocus-genotype combination is shown in Figure 3. In the model of FGF19 rs948992 – FGF21 rs11665896 – FGF23 rs11063118, (TC) – (TG) – (TT) presented the high-risk genotype combination, (CC) – (TT) – (CC) and (TT) – (TT) – (CC) presented the low-risk genotype combinations (Figure 3).

**TABLE 7:** The best model for predicting the aggression in AD.

**Best model^*a*^**	**Training accuracy (%)**	**Testing accuracy (%)**	**CVC**	**χ^2^**	***p***	**95% CI**
1	53.32	50.78	9/10	0.23	0.629	0.94 (0.76, 1.18)
1, 2	54.09	50.32	8/10	1.39	0.237	0.87 (0.70, 1.09)
1, 2, 3	56.85	52.66	9/10	9.89	0.0017	1.44 (1.15, 1.80)
1, 2, 3, 4	58.19	51.43	6/10	0.568	0.451	1.08 (0.88, 1.33)
1, 2, 3, 4, 5	59.33	53.88	10/10	0.213	0.644	0.95 (0.75, 1.19)

*^*a*^Number 1–5 represented FGF19 rs948992, FGF21 rs11665896, FGF23 rs11063118, FGF23 rs7955866, and FGF21 rs11665841, respectively.*

## Discussion

In the present study, we attempted to evaluate the association between AD-related aggression and genetic variants of the FGF19 subfamily members including FGF19, FGF21, and FGF23 genes in a Chinese population. No significant differences were found of frequencies of allelic distribution of 5 SNPs in three loci of FGFs between AD and HC. We found that the aggression level of the AD subjects was higher than that of the HC group. Our results had also shown that the interaction of FGF21 rs11665896 GG × FGF19 rs948992 TC presented the high-risk genotype combination associated with AD, and FGF21 rs11665896 TT × FGF19 rs948992 TT presented the low-risk genotype combinations of AD. In addition, FGF19 rs948992 TC × FGF21 rs11665896 TG × FGF23 rs11063118 TT presented the high-risk genotype combination of aggression associated with AD.

The behavioral impairments related to alcohol have been reported in numerous studies about impulsivity, aggression, depression, suicidal behavior, and other mental disorders (Tobore, 2019). Alcohol-related aggression was a common violent behavior in individuals with AD, it was considered a brain disfunction with cognitive disruption (Heinz et al., 2011). It has been suggested that alcohol could disrupt the crucial contents of social information and further engendered aggressive behavior under mis-interpretation (Beck and Heinz, 2013; Parrott and Eckhardt, 2018). But this association showed a controversial result with the development of alcohol exposure. Some studies suggested that increasing alcohol consumption was associated with violent behaviors including aggression (Stappenbeck and Fromme, 2010; Sacco et al., 2015). In an animal model, alcohol-related aggression increased with elevated doses of alcohol, but the dose-dependent association might be disrupted by higher doses (Weerts et al., 1993). Other studies reported that alcohol-related aggression did not happen much in individuals with AD (Murphy et al., 2005; Beck and Heinz, 2013). Thus, acute and excessive alcohol consumption seemed to more likely engender aggressive behavior (Fals-Stewart, 2003). In our study, higher MAST scores presented a higher risk of aggression in individuals with AD.

The studies about the genetic link between the FGF21 gene and alcohol intake increased gradually. It has been demonstrated that rs838133 and rs838145 were associated with higher carbohydrate intake, which also may affect alcohol intake (von Holstein-Rathlou and Gillum, 2019). The two SNPs in this present study, including rs11665896 and rs11665841, only rs11665896 was confirmed to influence carbohydrate metabolic (Ruiz-Padilla et al., 2019). Another study attempted to assess the association between rs11665841 and carbohydrate metabolism, but no significant association was found (Zhang et al., 2012). Although FGF19 mRNA overexpression was associated with alcohol-related decreases (Zhang et al., 2013), whether the SNPs of FGF19 including rs948992 were related to alcohol intake remains not clear. In this study, we found the interaction of FGF19 rs948992 TC × FGF21 rs11665896 GG presented the high-risk genotype combination associated with AD.

MicroRNAs constitute a growing class of non-coding RNAs that suppress target gene protein translation by combining with 3′ UTR (Bartel, 2004). Rs948992 is located on 3′ UTR of FGF19 gene, and rs1166584 and rs11665896 are both located about 500 bp downstream at 3′ end of the FGF21 gene, based on dbSNP database. The T allele of rs11665896 carriers consumed higher amounts of carbohydrates compared to carrying the GG or GT genotypes (Ruiz-Padilla et al., 2019), and alcohol in circulation was delayed in the G allele carriers compared to the T carriers, which means that the metabolic capability of GG genotype for alcohol may be weaker than the TT genotype. In addition, the two SNPs are located at the 3′UTR region, in which target sites for miRNAs are located and their genetic variants were associated with genotype alteration (Zhang et al., 2012). Speculatively, the change of allele exchange resulting from these loci could affect miRNA binding, perhaps reducing FGF19 and FGF21 transcription, as it has been shown for other genes (Sethupathy and Collins, 2008; Moszynska et al., 2017). In the present study, the interaction combination of FGF19 rs948992 TC × FGF21 rs11665896 TG × FGF23 rs11063118 TT showed a significant correlation with aggression associated with AD.

Some limitations of the present study should be noted. Firstly, the age and the years of education were not well-matched between the HC and AD subjects. The age and education years may have effects on aggression, although the conclusions about the effects of age and education years on aggression are inconsistent in previous studies. A recent study suggested that physical aggression declined with age in boys instead of girls, but indirect aggression increased with age in girls, such as anger and hostility (Tsorbatzoudis et al., 2013). Another study suggested that age had bi-directional moderating effects on aggression based on the levels of reactive aggression (Smeets et al., 2017). Generally for male drinkers, daily alcohol intake increases with age but declines in those with more education (Li et al., 2014). Perpetrators of peer aggression were reported to be associated with non-completion of secondary school (Moore et al., 2015) and aggression negatively predicted higher education (Rabinowitz et al., 2020). Although the statistical analysis of ANCOVA was performed with age and education years as covariates to weaken the influence of age and education year differences on aggression between the HC and AD groups, age and education year differences might still possibly cause the observed difference in the aggression of HC and AD groups in the present study. Secondly, more variations of these three genes, including tags and functional variations should be genotyped to obtain more information. Finally, the information of SNPs on FGF 23 in AD and aggression is rare, and needs to be further investigated.

## Conclusion

The aggression increases significantly associated with the level of AD in the AD subjects. The interaction of FGF19 rs948992 TC × FGF21 rs11665896 GG presented the high-risk genotype combination predicting the high level of AD. In addition, the interaction of FGF19 rs948992 TC × FGF21 rs11665896 TG × FGF23 rs11063118 TT presented the high-risk genotype combination predicting the high level of aggression in AD patients.

## Data Availability Statement

The raw data supporting the conclusions of this article will be made available by the authors, without undue reservation.

## Author Contributions

JH and HX conceived and designed the research. JX, FeW, FaW, FY, MLi, MLo, LW, and JH performed the experiments and wrote the manuscript together. HL and YF supplied the important and thoughtful advice and performed the experiments. LC, YL, and WL performed the statistical analysis and supplied much technology and fund assistant. FeW performed the experiments and wrote the manuscript, and made an important contribution to the work. All authors discussed the drafting of the manuscript. HX edited the last version of the manuscript.

## Conflict of Interest

The authors declare that the research was conducted in the absence of any commercial or financial relationships that could be construed as a potential conflict of interest.

## Publisher’s Note

All claims expressed in this article are solely those of the authors and do not necessarily represent those of their affiliated organizations, or those of the publisher, the editors and the reviewers. Any product that may be evaluated in this article, or claim that may be made by its manufacturer, is not guaranteed or endorsed by the publisher.

## Footnotes

1
https://www.ncbi.nlm.nih.gov/snp/rs948992

2
http://analysis.bio-x.cn/myAnalysis.php
